# 5α-reductase inhibitors impact prognosis of urothelial carcinoma

**DOI:** 10.1186/s12885-020-07373-4

**Published:** 2020-09-11

**Authors:** Chien-Sheng Wang, Ching-Chia Li, Yung-Shun Juan, Wen-Jeng Wu, Hsiang-Ying Lee

**Affiliations:** 1 Department of Urology, Kaohsiung Medical University Hospital, Kaohsiung, Taiwan; 2 Department of Urology, School of Medicine, College of Medicine, Kaohsiung Medical University, No.100, Shiquan 1st Rd., Sanmin Dist, Kaohsiung City, 807 Taiwan; 3 Kaohsiung Municipal Ta-Tung Hospital, Kaohsiung, Taiwan; 4 Regenerative Medicine and Cell Therapy Research Center, Kaohsiung Medical University, Kaohsiung, Taiwan; 5 Graduate Institute of Clinical Medicine, College of Medicine, Kaohsiung Medical University, Kaohsiung, Taiwan

**Keywords:** 5α-reductase inhibitors (5-ARIs), Bladder cancer, Upper tract urothelial carcinoma, Androgen receptor (AR), Finasteride, Dutasteride

## Abstract

**Background:**

5α-reductase inhibitors (5-ARIs) inhibit the pathway of converting the testosterone to dihydrotestosterone and are widely used in benign prostatic hyperplasia patients. Since androgen receptor activation may play a role in urothelial tumorigenesis, we conducted this retrospective cohort study to determine whether 5α-reductase inhibitors (5-ARIs) administration is associated with bladder cancer mortality, bladder cancer recurrence and upper tract urothelial carcinoma mortality, using the Taiwan National Health Insurance database.

**Methods:**

The data of this retrospective cohort study were sourced from the Longitudinal Health Insurance Database of Taiwan, compiled by the Taiwan National Health Insurance database from 1996 to 2010. It consists of 18,530 men with bladder cancer, of whom 474 were 5-ARIs recipients and 4384 men with upper tract urothelial carcinoma, of whom 109 were 5-ARIs recipients. Propensity Score Matching on the age and geographic data was done at the ratio of 1:10. We analyzed the odds ratios (OR) and 95% confidence interval (CI) of the risk of bladder cancer death, bladder cancer recurrence rate and upper tract urothelial carcinoma related death by the 5-ARIs administration.

**Results:**

Those who received 5-ARIs showed a lower risk of bladder cancer related death compared to nonusers in multivariable adjusted analysis (OR 0.835, 95% CI 0.71–0.98). However, there was no significant difference in the bladder cancer recurrence rate (OR 0.956, 95% CI 0.82–1.11) and upper tract urothelial carcinoma related mortality in multivariable adjusted analysis (OR 0.814, 95% CI 0.6–1.1).

**Conclusions:**

Patients who receive 5-ARIs have lower bladder cancer related mortality compared to those who don’t. 5-ARIs may prove to be a viable strategy to improve bladder cancer outcomes.

## Background

Bladder cancer is the ninth most common cancer type in the world [1]. Most of the bladder cancers are superficial and can be treated with transurethral resection of bladder tumor (TURBT) often combined with intravesical instillation of hemotherapeutic agents or bacillus Calmette–Guérin (BCG). Approximately 25% of bladder cancer infiltrates the muscle layer of the bladder wall and in these cases, radical cystectomy combined with chemotherapy are often needed [2]. Although the 5 years survival rate of non-muscle invasive bladder cancer (NMIBC) is more than 88%, up to 70% of NMIBC recur after initial treatment and 10–20% may progress into muscle-invasive bladder cancer [3]. The high recurrence rate and the need for lifelong surveillance make it a considerable burden on public health insurance.

Unlike bladder cancer, upper urinary tract carcinoma (UTUC), including renal pelvis and ureter diseases, are uncommon and account for only 5–10% of urothelial carcinoma (UC) in Western countries [4] but account for 10–25% of all UC in Taiwan. The standard therapy for UTUC is Nephroureterectomy with bladder cuff excision (radical nephroureterectomy). Patients with high-stage disease often have poor prognosis. The 5-year survival rate is < 50% for tumor (T) stage T2/3 and < 10% for patients with T4 UTUC [5].

The major risk factors for bladder cancer include exposure to cigarette and industrial chemicals, which could explain why bladder cancer is more common in men than in women. However, a previous study has shown that gender remained a significant risk factor with exposure to smoking and industrial chemicals as control variables [6].

An animal model conducted by Okajima et al. has proposed that androgen receptor (AR) signals may be related to bladder cancer development. They discovered that by blocking the androgen pathway with androgen deprivation therapy may reduce the risk of bladder cancer [7]. Izumi et al. has reported that androgen deprivation therapy (ADT) may have a inhibitory effect on the intravesical recurrence of bladder cancer [8]. As a result, androgen receptor activation may play a role in urothelial tumorigenesis and progression, which makes androgen receptor inactivation a potentially viable urothelial cancer treatment [9].

The enzyme 5α-reductase, which is mainly expressed in the prostate, converts the testosterone to dihydrotestosterone, the main activator of androgen signaling. 5α-reductase inhibitors (5-ARIs) such as Finasteride and Dutasteride inhibit the pathway and therefore are widely used in benign prostatic hyperplasia patients. Since androgen receptor activation may play a role in urothelial tumorigenesis, we can presume that 5-ARIs would be beneficial to patients with bladder cancer. The Prostate, Lung, Colon, and Ovarian (PLCO) trial, a large population-based randomized trial designed and sponsored by the National Cancer Institute (NCI), has shown that patients who received the finasteride or dutasteride may have a reduced incidence of bladder cancer compared to nonusers [10]. Similarly, a few studies have also described how 5-ARIs may affect the bladder cancer prognosis [11–13]. However, by comparing groups between placebo, doxazosin, finasteride, or combination of both treatments, Niranjan et al. discovered a controversial finding that there was no observable relationship between finasteride and bladder cancer incidence [14].

In this study, we performed a population based retrospective cohort study to determine whether 5-ARIs usage affects bladder cancer incidence and recurrence, using the Taiwan National Health Insurance (NHI) database. We’ve also compared the effect on Upper Tract Urothelial Carcinoma and presumed that the prognosis would be better among men who received 5-ARIs compared to nonusers.

## Methods

### Study design and data source

This study was approved by our institutional review board (KMUHIRB-EXEMPT(I)-20180044) in Kaohsiung Medical University Chung-Ho Memorial Hospital, Taiwan. Our data were collected from the Longitudinal Health Insurance Database of Taiwan, during the time period of 1998 to 2010. The Longitudinal Health Insurance Database of Taiwan contains diagnoses and procedures recorded at hospital contacts at Taiwan health care units. Since 98% of all patients are covered by our insurance system, the database was presumed to include most of the outpatients and admission medical records in Taiwan. Because 5-ARIs are available only by prescription in Taiwan, they are comprehensively recorded by the database. This study was supervised by the review board of Kaohsiung Medical University Hospital, Kaohsiung, Taiwan.

### Study population

This is a retrospective design study. Patients with cancer are defined as (1) male patients (2) aged over 18 years old (3) assigned by urologists (4) the ICD-9-CM (The International Classification of Diseases, Ninth Revision, Clinical Modification) diagnostic code 189 for bladder cancer and ICD-9-CM diagnostic code 188 for Upper tract urothelial carcinoma. Patients younger than 18 years old, cases with incomplete demographic data, female patients, and those who were ever diagnosed of other cancer before index time were excluded. Available data included date of cancer diagnosis, primary and secondary treatment (surgery vs other), and the date and cause of death. Information on medication exposure to 5-ARIs was also obtained from the Longitudinal Health Insurance Database of Taiwan under the Anatomical Therapeutic Chemical code G04CB01. Those who have exposure dose equal to and greater than 28 defined daily dose (DDD) were included. Surgical procedures were identified by procedure codes 78008C for transurethral resection of the bladder tumor; 78010C for partial cystectomy; 78011B, 78039B, 78040B, 78012B, 78013B, 78014B, 78041B, 78042B, 78043B, 78044B, 78045B, 78046B for Radical cystectomy. Propensity Score Matching on the age and geographic data was applied at the ratio of 1:10. The comorbidities were defined by ICD-9 diagnostic codes, including diabetes mellitus (ICD-9 diagnostic codes 250), hypertension (401–405), chronic kidney disease (585), and hyperlipidemia (272.0–272.4).

### Statistical analysis

χ2 or Fisher’s exact test were used to assess the differences between categorical parameters. We used propensity score matching to reduce the bias of confounding variables. We analyzed the odds ratios (OR) and 95% confidence interval (CI) of the risk of bladder cancer death, recurrence rate, and upper tract urothelial carcinoma death among the use of 5-ARIs using Cox regression adjusted for age, and comorbidities including diabetes mellitus, hypertension, chronic kidney disease and hyperlipidemia. Follow up continued until death, emigration or the common closing date of December 31, 2010, whichever came first. All reported ORs in this article are multivariable adjusted, unless otherwise stated. Statistical significance was set at *P* < 0.05. SPSS 20.0 (SPSS Inc., Chicago, IL) was used for all statistical analyses.

## Results

Among the study population of 18,530 men with bladder cancer, 474 were 5-ARIs recipients. Propensity Score Matching on the age and geographic data was applied at the ratio of 1:10 (Table 1). The number of deaths caused by bladder cancer in the 5-ARIs group was 163 (34.39%), and 1873 (39.51%) in the control group. 185 (44.90%) participants in the 5-ARIs group experienced tumor recurrence, in contrast with the 1851 (44.93%) in the control group.

**Table 1:** Basic characteristics between 5-ARIs User and comparison groups with Bladder Cancer (*n* = 5214)

	5-ARIs User, n (%)	None User, n (%)	*P*-value
Case No.	474	4740	
Variables			
Age (Mean ± SD)	76.47 (±7.90)	76.60 (±8.51)	0.733
Age category (n, %)			
< 65	42 (8.86)	466 (9.83)	
65–74	153 (32.28)	1387 (29.26)	
> 75	279 (58.86)	2887 (60.91)	
CCI score (Mean ± SD)	4.23 (±2.18)	4.22 (±2.22)	0.959
CCI score category			
0 ~ 3	220 (46.41)	2203 (46.48)	
4 ~ 7	202 (42.62)	2037 (42.97)	
8+	52 (10.97)	500 (10.55)	
Comorbidity			
Hypertension	258 (54.43)	2546 (53.71)	0.765
Hyperlipidemia	88 (18.57)	842 (17.76)	0.664
Diabetes mellitus	114 (24.05)	1121 (23.65)	0.845
Chronic Kidney Disease	36 (7.59)	378 (7.97)	0.771

*CCI* Charlson Comorbidity Index

Among the study population of 4384 men with upper tract urothelial carcinoma, 109 were 5-ARIs recipients. Propensity Score Matching was applied to the age and geographic data at the ratio of 1:10 (Table 2). The number of deaths caused by bladder cancer was 47 (43.12%) in the 5-ARIs group and 525 (48.17%) in the control group.

**Table 2:** Basic characteristics between 5-ARIs User and comparison groups with Upper Tract Urothelial Carcinoma (*n* = 1199)

	5-ARIs User, n (%)	None User, n (%)	P-value
Case No.	109	1090	
Variables			
Age (Mean ± SD)	75.42 (±7.56)	75.75 (±7.62)	0.668
Age category (n, %)			
< 65	5 (4.59)	87 (7.98)	
65–74	44 (40.37)	366 (33.58)	
> 75	60 (55.05)	637 (58.44)	
CCI score (Mean ± SD)	4.84 (±2.55)	4.68 (±2.40)	0.501
CCI score category			
0 ~ 3	38 (34.86)	390 (35.78)	
4 ~ 7	55 (50.46)	547 (50.18)	
8+	16 (14.68)	153 (14.04)	
Comorbidity			
Hypertension	57 (52.29)	553 (50.73)	0.756
Hyperlipidemia	24 (22.02)	227 (20.83)	0.770
Diabetes mellitus	31 (28.44)	292 (26.79)	0.711
Chronic Kidney Disease	17 (15.60)	169 (15.50)	0.980

*CCI* Charlson Comorbidity Index

**Fig. 1:**
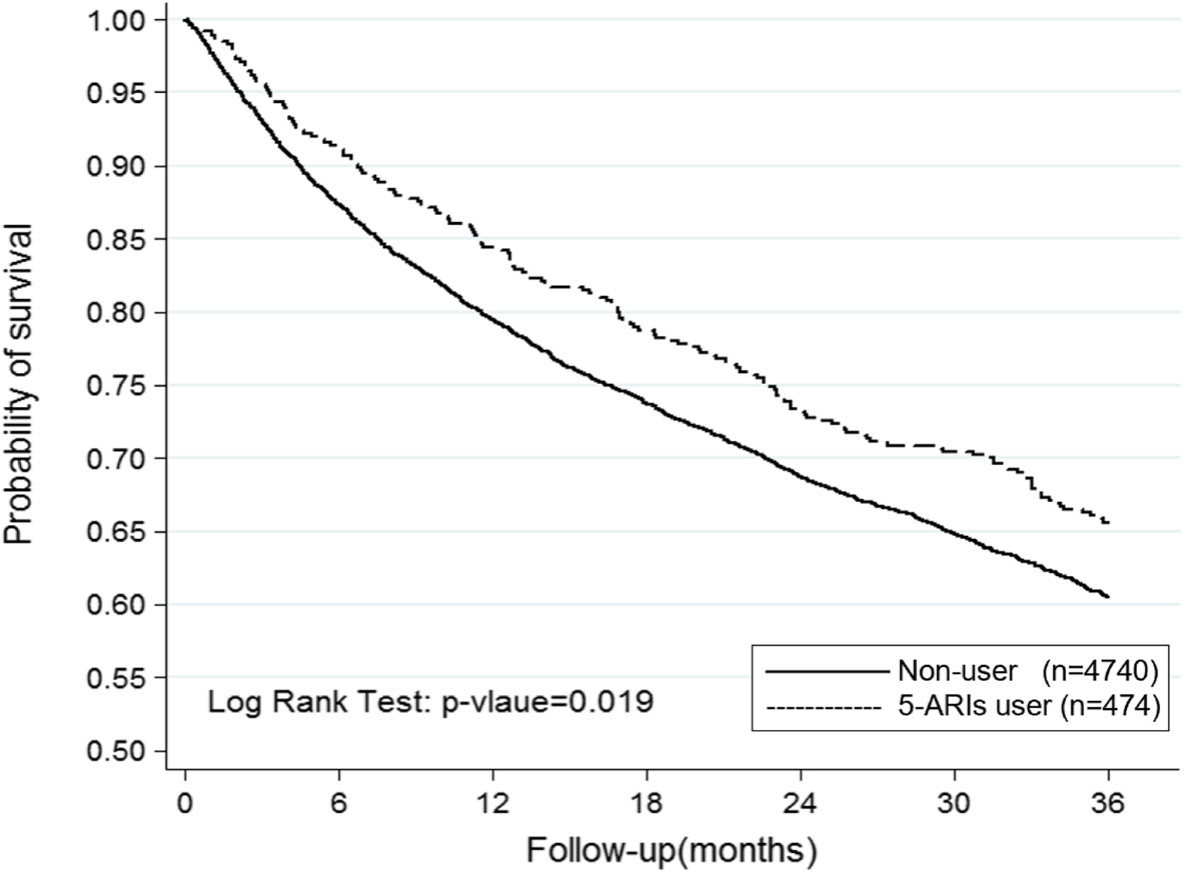
Kaplan-Meier estimate of Bladder Cancer related mortality in patients who received 5α-reductase inhibitor and nonuser

**Fig. 2:**
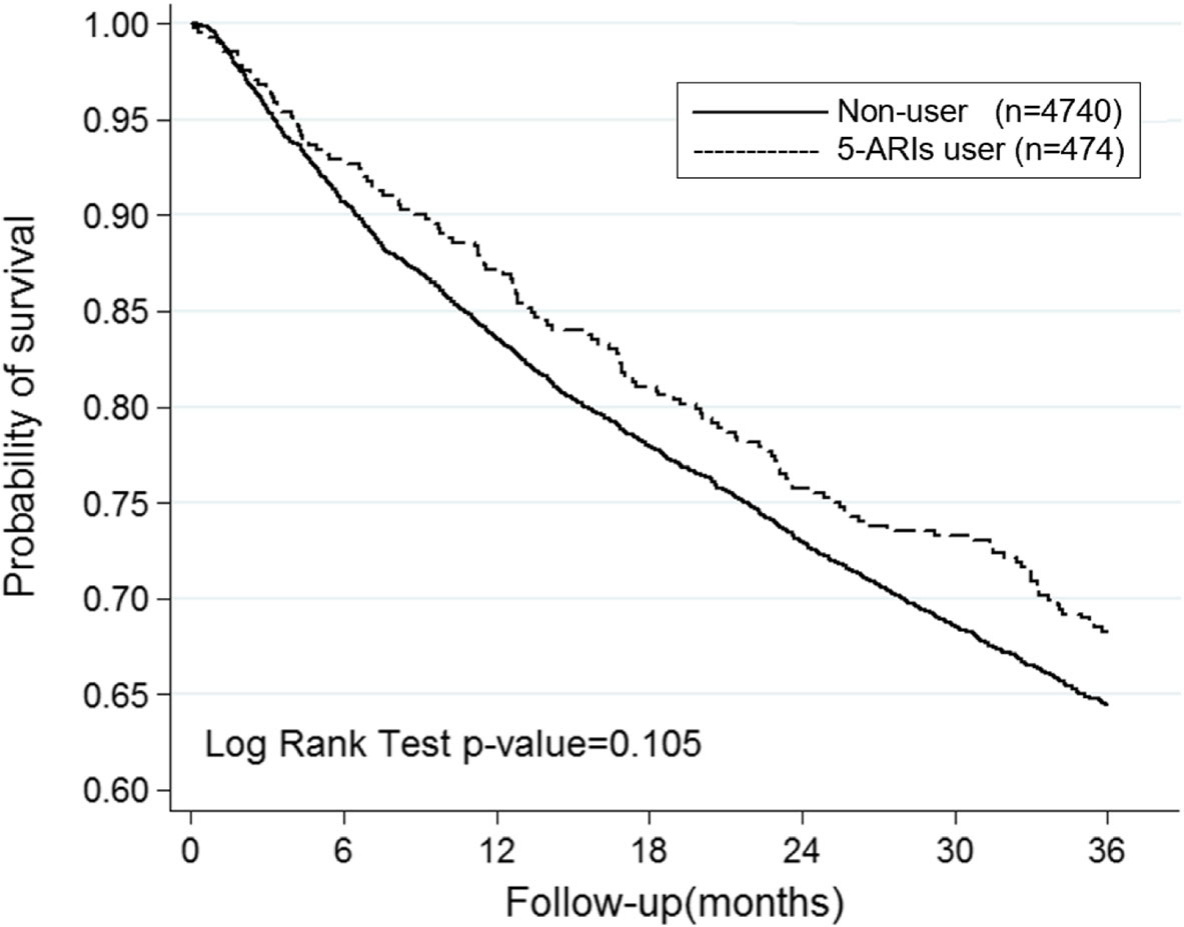
Kaplan-Meier estimate of Upper Tract Urothelial Carcinoma related mortality in patients who received 5α-reductase inhibitor and nonuser

**Fig. 3:**
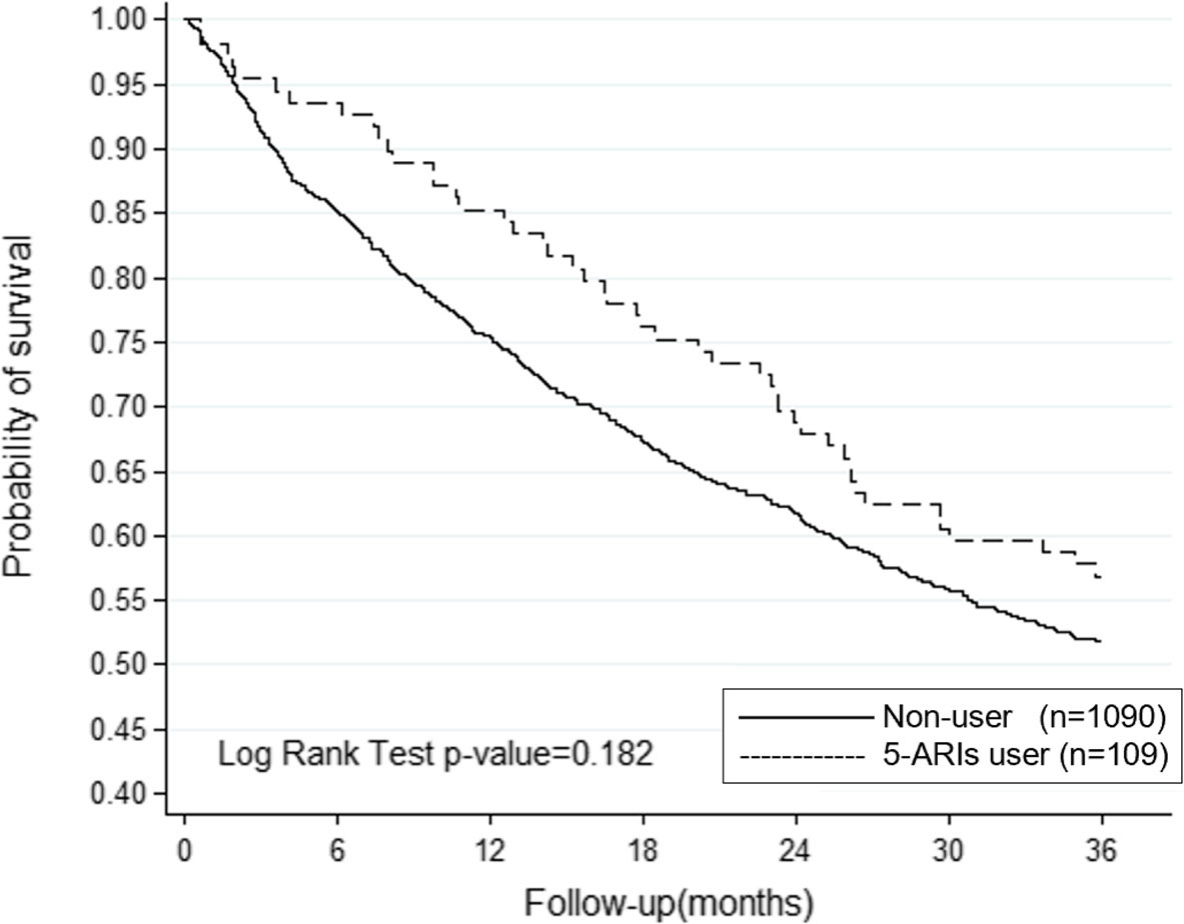
Kaplan-Meier estimate of Bladder Cancer related recurrence rate in patients who received 5α-reductase inhibitor and nonuser

Compared to nonusers, those who received 5-ARIs showed a lower risk of bladder cancer death in multivariable adjusted analysis (OR 0.835, 95% CI 0.71–0.98, Table 3 & Fig. 1). However, there was no significant difference between men who received 5-ARIs and nonusers in bladder cancer recurrence rate (OR 0.956, 95% CI 0.82–1.11, Table 4 & Fig. 2). As for the upper tract urothelial carcinoma group, there was no significant difference between men who received 5-ARIs and nonusers in mortality rate in multivariable adjusted analysis (OR 0.814, 95% CI 0.6–1.1, Table 3 & Fig. 3).

**Table 3:** Conditional logistic regression measured odds ratio and 95% confidence intervals of mortality rate

Characteristics	Crude			Adjusted		
	OR	95%CI	*P*-value	OR	95%CI	*P*-value
5-ARIs						
Bladder Cancer	**0.826**	**0.7–0.97**	**0.018**	**0.835**	**0.71–0.98**	**0.019**
UTUC	0.817	0.61–1.1	0.179	0.814	0.6–1.1	0.182

*5-ARIs* 5α-reductase inhibitors, *UTUC* Upper Tract Urothelial Carcinoma, *OR* odds ratio, *CI* confidence interval, *Adjusted OR* Adjusted for age and comorbidities

**Table 4:** Conditional logistic regression measured odds ratio and 95% confidence intervals of recurrence rate

Characteristics	Crude			Adjusted		
	OR	95%CI	*P*-value	OR	95%CI	*P*-value
5-ARIs						
Bladder Cancer	0.960	0.83–1.12	0.109	0.956	0.82–1.11	0.105

*5-ARIs* 5α-reductase inhibitors, *OR* odds ratio, *CI* confidence interval, *Adjusted OR* Adjusted for age and comorbidities

## Discussion

Our report is to clarify the association between 5-ARIs and the survival of bladder cancer and upper tract urothelial carcinoma. The mortality rate related to the bladder cancer in the 5-ARIs group (34.39%) was significantly lower compared to the control group (39.51%). (Fig. 1) The data from this study were collected from nationwide databases which provided large case number and accurate information on cancer diagnosis, drug purchases and cancer related deaths. The exact drug use history enabled us to evaluate the relationship between medication use and cancer related death.

5-ARIs are widely used in patient with benign prostate hyperplasia since it can reduce prostate volume and improve urinary outflow obstruction. But they can also lower the serum prostate-specific antigen (PSA) value by approximately 50%, which may lead to a delay in the diagnosis of prostate cancer [15]. Some animal studies confirmed that 5a-reductase is also expressed in urothelial cancer cells and dihydrotestosterone may affect the growth of those cells [7–9]. As a result, 5-ARIs may be beneficial to patients with urothelial cell cancer.

As shown in our study, men using 5-ARIs have lower bladder cancer mortality rate compared to nonusers. Researches regarding the molecular mechanisms of bladder cancer carcinogenesis has outlined the potential role the androgen receptor (AR) plays [16]. The exact mechanism remains unclear, but some studies have suggested that AR signaling is associated with deoxyribonucleic acid (DNA) breaks, chromosomal rearrangements, and the suppression of the Uridine 5′-diphospho-glucuronosyltransferase, which play a role in carcinogens elimination [17, 18]. Studies involved mouse models have demonstrated that androgen deprivation therapy seems to decrease the incidence of tumorigenesis [19]. Ding et al. has also suggested that the activation of androgen receptor (AR) may promote bladder cancer cell proliferation and migration [20]. Therefore, using these findings to prevent bladder cancer seems promising.

Finasteride, a 5-ARIs widely used for benign prostate hyperplasia, was proposed to be associated with a decreased risk of developing bladder cancer (hazard ratio: 0.634; 95% confidence interval, 0.493–0.816; *p* = 0.0004), controlling for age and smoking, in the Prostate, Lung, Colorectal, and Ovarian cancer screening (PLCO) trial [10]. However, it is a retrospective, observational study and the use of finasteride was self-reported, not confirmed by pill counts or prescription analysis; the results should be interpreted with caution.

Similar results were also obtained in two other case-control studies. Chen et al. found that 5α-reductase genomic alternations were observed in 29% of bladder cancer patients and they discovered that Finasteride has a direct inhibition effect on bladder cancer cell growth [13]. Shiota et al. conducted a cohort study involving 228 Japanese under 5-ARIs or androgen deprivation therapy and suggested that these men were at a lower risk of intravesical recurrence and progression to muscle invasive bladder cancer [21]. These studies suggest that 5-ARIs may have a potential therapeutic effect in preventing or even treating bladder cancer.

In our country, 5-ARIs is mainly prescribed to those with benign prostate hyperplasia with lower urinary tract symptoms. These patients tend to receive abdominal echo or cystoscopy more often, which increases bladder tumor detection rate and may generate a lead time bias. However, a study conducted by Ville et al. has compared the bladder cancer specific mortality rate between 5-ARIs and α-blockers, a drug group that may also be affected by the same lead time bias with different medication mechanism. The risk reduction was only discovered in the 5-ARIs group, which indicated that lead time bias could not fully explain the decreased mortality rate [22].

Contrary to the bladder cancer group, there was no significant difference in the mortality rate of patients with upper tract urothelial carcinoma who received 5-ARIs to nonusers. But since the case number was relatively small compared to the bladder cancer group, further research is required to understand the true therapeutic implications.

The main limitation of the study is its retrospective nature and the 5-ARIs use was not randomized. These needs comprehensive in vitro, in vivo and clinical study in the future for further verification and confirmation of these findings. The demographic data available in our study was analyzed with permitted propensity score, which may improve our study strength. However, we did not collect the data of other risk factors for urothelial cancer, such as smoking status, workers exposed to arylamines and dye industries, or other medication exposure such as Chinese herbal supplements. Therefore, it was impossible to adjust for these factors in our analysis.

## Conclusion

Our study aims to evaluate the role of 5-ARIs in the mortality and recurrence rate of bladder cancer and upper tract urothelial carcinoma. Based on our study, 5-ARIs could potentially reduce the mortality of bladder cancer, which also supports the theory that androgens may suppress bladder cancer progression. Such risk reduction was not observed in men with upper tract urothelial carcinoma. Although these findings are limited by the study’s retrospective design and a lack of data on confounders such as smoking history, administering 5-ARIs may still prove to be a possible strategy to improve bladder cancer outcomes.

## Data Availability

The datasets used and/or analysed during the current study are available from the corresponding author on reasonable request.

## Abbreviations

5-ARIs: 5α-reductase inhibitors
OR: Odds ratios
CI: Confidence interval
AR: Androgen receptor
TURBT: Transurethral resection of bladder tumor
ADT: Androgen deprivation therapy
NCI: National Cancer Institute
ICD-9-CM: The International Classification of Diseases, Ninth Revision, Clinical Modification
PSA: Prostate-specific antigen
DNA: Deoxyribonucleic acid
UTUC: Upper Tract Urothelial Carcinoma
CCI: Charlson Comorbidity Index
NMIBC: Non-muscle invasive bladder cancer
UC: Urothelial carcinoma
